# A Rare Occurrence of Scleromalacia Perforans With Juvenile Rheumatoid Arthritis: A Case Report

**DOI:** 10.7759/cureus.27520

**Published:** 2022-07-31

**Authors:** Mayur B Wanjari, Tejaswee Lohakare, Ashwini Potdukhe, Pratiksha K Munjewar, Vaishnavi V Kantode

**Affiliations:** 1 Research, Jawaharlal Nehru Medical College, Datta Meghe Institute of Medical Sciences (Deemed to be University), Wardha, IND; 2 Child Health Nursing, Srimati Radhikabai Meghe Memorial College of Nursing, Datta Meghe Institute of Medical Sciences (Deemed to be University), Wardha, IND; 3 Medical Surgical Nursing, Srimati Radhikabai Meghe Memorial College of Nursing, Datta Meghe Institute of Medical Sciences (Deemed to be University), Wardha, IND

**Keywords:** deformities, eye, antibiotics, scleromalacia perforans, chronic juvenile rheumatoid arthritis

## Abstract

Here, we report a case of a nine-year-old boy with chronic juvenile rheumatoid arthritis (JRA) leading to scleromalacia perforans (SP). We rarely see SP with fixed deformities of rheumatoid arthritis in the hands, but not as a starting point of the disease. He reported eye manifestations associated with JRA on further investigation and inquiry. The right globe was preserved on presentation, and the left was perforated. He lost sight in his left eye when he was treated with antibiotics and visited various physicians in his native region.

## Introduction

Juvenile rheumatoid arthritis (JRA) is the most common disease condition in children. JRA is a primary subtype based on the manifestation at disease onset and is identified as pauciarticular onset, polyarticular onset, and systemic onset. The prevalence rate of JRA is 100,000 per year among children aged 15 years or younger. Scleromalacia perforans (SP) is an uncommon type of anterior scleritis that appears as dark blue seen through a thin sclera [1]. A rare type of necrotizing anterior scleritis is distinguished by increasing sclera thinning but neither pain nor redness [2].

## Case presentation

A case of nine-year-old boy presented to the outpatient department (OPD) with a complaint of pain and dryness in the left eye for three months, along with progressive loss in vision in the affected eye for one month. Furthermore, he also experienced stiffness and pain in the joints for the past two months. A few days prior to his OPD visit, he started experiencing similar symptoms of pain, blurred vision, and photophobia in his right eye.

His parents reported that the child had severe pain in the left eye with a gritty sensation. The pain score was 7/10 as assessed with the visual analog scale (VAS). The child’s pain was accompanied by dryness of the eye with a history of pus discharge from the left eye, and progressive vision loss was reported. He visited various hospitals in his native region. He was treated with antibiotics by doctors for 15 days. He complained of progressive vision loss after this treatment when he came to our OPD.

**Figure 1:**
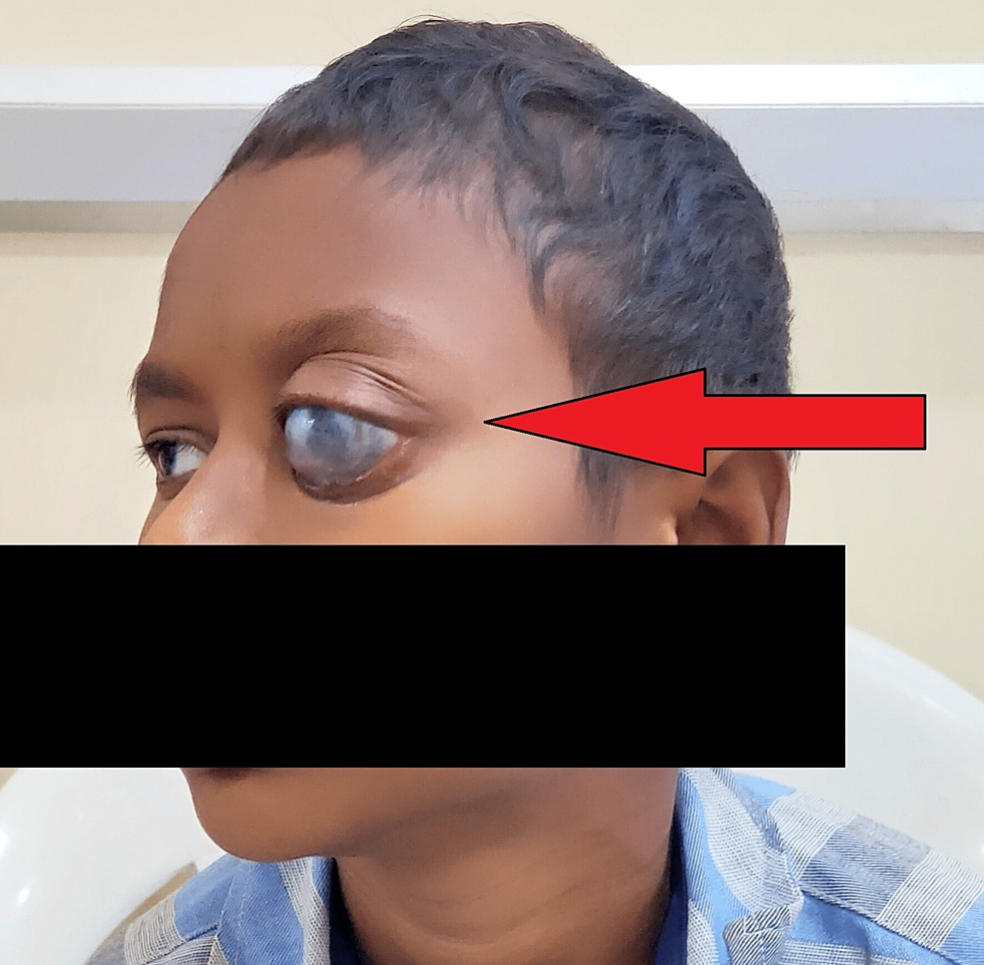
Left eye shows melted left cornea and inferior peripheral ulceration

On clinical examination, the ophthalmologist revealed that the patient’s eye did not respond to light perception. A slit-lamp examination of the left eye revealed a melted cornea and inferior peripheral ulceration. It showed a melted left cornea, and his left eye had inferior peripheral ulceration. There was thinning in the sclera at the lower temporal quadrant with visible uveal tissue. The peripheral cornea was also thin in the lower temporal quadrant (Figure 1).

The patient was treated with methylprednisolone 500 mg/once a day, methotrexate 10 mg/week, folic acid supplementation, and a proton pump inhibitor. After three months of treatment, steroids were tapered to 5 mg/day, and hydroxychloroquine 400 mg/day was initiated. The patient exhibited a satisfactory response to this treatment.

## Discussion

The is a rare report of JRA leading to SP. Lamba et al. concerned various manifestations and underscored the necessity for earlier identification and remedy [3]. RA is a complex condition that may impact the eyes. Ophthalmic manifestations of RA reported in the literature include keratoconjunctivitis, episcleritis, scleritis, peripheral ulcerative keratitis, and retinal vasculitis [4].

The incidence of SP in a patient through a 10-year history of joint pains was discussed by Wu et al. [5] similar to our case, who reported JRA intricacies preceding SP. According to Watson and Hayreh (1976), scleritis is a severe erythrogenic disease characterized by edema and inflammatory cell infiltration of the sclera, which frequently manifests as pain and redness [6]. It is mainly prevalent during the fifth decade but is more common in the fourth to sixth decades [6]. McCluskey and Wakefield noted that it is bilateral in 40% of the cases [7]. In this case, the patient loses his left eye within a month; the objective of managing a patient with scleritis is to identify the lethal systemic etiology, manage and stabilize ocular and systemic inflammation, make stable, and prevent scleral melt.

Watson and Hayreh classified scleritis into anterior and posterior types based on the anatomic classification of the disease [8]. The most typical type of scleritis is anterior scleritis [9,10]. It is essential to address the ocular manifestations due to the possibility of permanent damage. Peripheral ulcerative keratitis, peripheral corneal thinning, and peripheral stromal keratitis are common complications of anterior scleritis. Posterior scleritis complications comprise exudative retinal detachment, optic disk edema, cystoid macular edema, and choroidal folds [11].

Scleritis may be associated with the local or systemic disease as stated by Galor and Thorne. Roughly, 40% of patients have an autoimmune condition, and 7% have infections [12]. SP is a potential autoimmune disease that shows as a black area of scleral thinness around the inflammatory tissue. Research shows a relation between scleritis and other systemic problems. In scleritis patients, the prevalence increased to nearly 50%, while 20%-30% of RA patients presented with subcutaneous nodules [10]. Patients with RA and scleritis are more likely to exhibit pulmonary and cardiac problems, compared to RA patients without scleritis. During a flare of RA, there is a chance of scleritis exacerbation [11].

The morbidity and mortality rates of patients with scleritis are higher. A 40%-50% of scleritis patients will die in three years if not treated with systemic medication. The mortality rate for patients with scleritis is 18% compared to the last three years. Extra-articular vasculitis is the leading cause of death. There is higher mortality associated with necrotizing scleritis [9]. Aggressive and systemic treatment is the most effective method of treating scleritis. Nonsteroidal anti-inflammation drugs can be used for the treatment of scleritis. Kanski and Bowling successfully treated two SP cases using adalimumab [13].

## Conclusions

Many extra-articular manifestations of RA are associated with ocular diseases. Efforts should be made between rheumatologists and ophthalmologists to evaluate patients with RA. Medical and surgical management are novel treatments with a good prognosis for the JRA. In this case, we treated the patient with medical management only, and the patient's prognosis was good. Hospitalization in the initial stage prevents further complications of the eye. Health check-ups and awareness programs in the school can prevent this type of disease in children early.
